# Germanium Based Field-Effect Transistors: Challenges and Opportunities

**DOI:** 10.3390/ma7032301

**Published:** 2014-03-19

**Authors:** Patrick S. Goley, Mantu K. Hudait

**Affiliations:** Advanced Devices & Sustainable Energy Laboratory (ADSEL), Bradley Department of Electrical and Computer Engineering, Virginia Tech, Blacksburg, VA 24061, USA; E-Mail: mantu.hudait@vt.edu

**Keywords:** germanium, heterogeneous integration, passivation, buffer, high mobility, gate stack, quantum well

## Abstract

The performance of strained silicon (Si) as the channel material for today’s metal-oxide-semiconductor field-effect transistors may be reaching a plateau. New channel materials with high carrier mobility are being investigated as alternatives and have the potential to unlock an era of ultra-low-power and high-speed microelectronic devices. Chief among these new materials is germanium (Ge). This work reviews the two major remaining challenges that Ge based devices must overcome if they are to replace Si as the channel material, namely, heterogeneous integration of Ge on Si substrates, and developing a suitable gate stack. Next, Ge is compared to compound III-V materials in terms of *p*-channel device performance to review how it became the first choice for PMOS devices. Different Ge device architectures, including surface channel and quantum well configurations, are reviewed. Finally, state-of-the-art Ge device results and future prospects are also discussed.

## 1. Introduction

**Figure 1.**
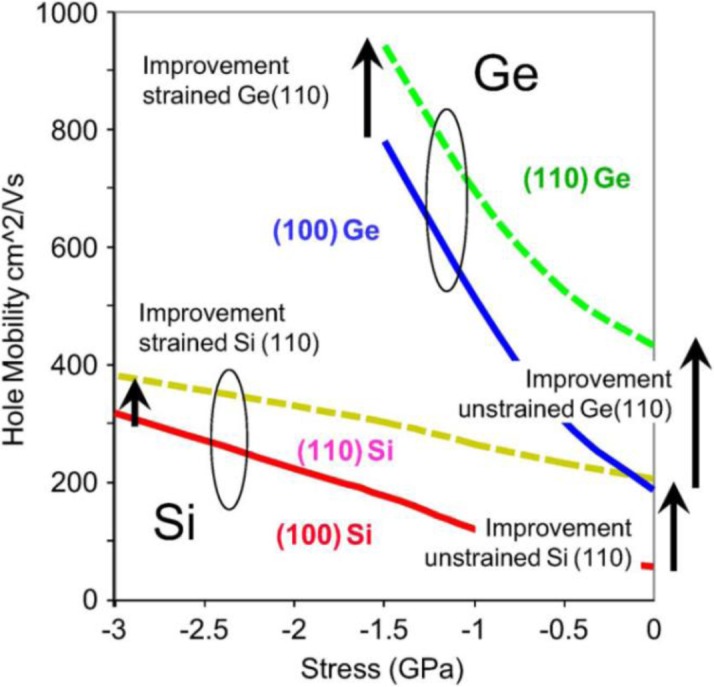
Hole mobility of Si and Ge as a function of stress and wafer orientation. Ge offers both better intrinsic hole mobility and better scalability compared to Si, reprinted with permission from [20]. Copyright 2012, IEEE.

The motivation to replace strained silicon (Si) with higher mobility channel materials in today’s metal-oxide-semiconductor field-effect transistors (MOSFETs) is well documented [1,2]. Saturation drive current (*I*_on_), a critical performance metric for FET devices, is intimately linked with carrier mobility. While this link has become less clear as devices are scaled down into quasi-ballistic and ballistic regimes, the general relation continues to hold [3]. In light of this relationship, future transistor scaling will require the introduction of new high mobility channel materials, including germanium (Ge) or III-V semiconductors. The higher carrier mobility offered by these materials compared to strained silicon presents a practical means to push *I*_on_ higher, even at lower supply voltages. On the other hand, even with the change in device architecture from planar to F in FET, continued scaling of today’s Si transistors is expected to provide only a few additional generations of technology. As seen in Figure 1, the hole mobility enhancement as a function of strain is much flatter in Si than for Ge. This suggests that if the challenges associated with Ge channel transistors can be overcome, the technology may continue to be scalable for decades.

The effort to move to higher mobility channel materials is complicated by the fact that it must, in a sense, be done twice, once for *n*-channel FETs (NMOS) and once for *p*-channel FETs (PMOS). The nature of complementary MOS logic (CMOS) requires comparably performing NMOS and PMOS devices working in tandem. Historically, NMOS transistors have outperformed PMOS since electron mobility (μ_e_) is generally significantly higher than hole mobility (μ_h_). Circuit designers have adapted to working with PMOS devices with about one third the performance of NMOS, so preferably the performance gap in the next generation of technology should be no larger than this [1]. Additionally, it is strongly favorable to use the same material system for both *n*-channel and *p*-channel devices (either Ge for both, or for example, InGaAs for both) since this substantially simplifies device processing [4,5]. Due to the exceptionally high μ_h_ of Ge, and the progress made in Ge based *p*-channel MOSFETs (pMOSFETs) [6–14] and *p*-channel quantum well FETs (pQWFETs) [2,15–19] over the last decade, there appears to be a consensus in the device research community and in industry that Ge offers the best option for PMOS devices [1,2,20]. In light of this, there is heightened incentive to develop Ge based NMOS devices that perform comparably. There are, however, significant additional engineering challenges to achieving this, such as overcoming Ge’s tendency for have a higher interface state density (*D*_it_) near the conduction band edge [21,22] and developing low resistance ohmic contacts to n-type Ge [23,24].

High mobility III-V materials present an alternative to Ge for future CMOS technology nodes. In III-V material systems, experimental outcomes have been the inverse of that seen with Ge—*i.e*., excellent III-V based NMOS devices have been demonstrated [25,26], whereas a comparably performing PMOS device using the same channel material is still elusive to date [1]. This is in large part due to the significant disparity in electron and hole mobility in III-V materials; with μ_e_ typically being at least several times larger than μ_h_ [1]. For this reason, it is not unlikely that a future CMOS technology node will involve co-integration of Ge based PMOS and III-V based NMOS devices together. Unfortunately, such a path would also entail finding ways to deal with the increased processing complexity that comes with having multiple material systems with different chemistries and thermodynamic stability on the same chip.

The above discussion has led to a choice of two paths, the dual-Ge path (for both NMOS and PMOS) or the III-V/Ge path (NMOS/PMOS). To be sure, there is also a possibility of a dual-III-V path; or it may be found the performance benefit of Ge and III-V over strained silicon (which is itself a moving target) fails to justify the increase in complexity and cost. The former seems the least likely of all alternatives, and the latter falls outside the scope of the present article. This review first summarizes the two major challenges that must be overcome to succeed in either path involving Ge: heterogeneous integration of Ge on Si, and developing a suitable gate stack. After this, a major challenge to the dual-Ge path, achieving low resistance ohmic contacts on n-type Ge, is briefly discussed. The basis for Ge’s selection over III-V for PMOS is also summarized. Finally, Ge device architectures and state-of-the-art results are reviewed. Many excellent review articles already exist for Ge based devices, with most focusing on the MOSFET architecture [2,11,27–30]. This review article presents recent advancements in the field of Ge based nanoscale MOSFETs and QWFETs.

## 2. Challenges

### 2.1. Heterogeneous Integration on Silicon

Due to low economic availability, any long-term widespread utilization of Ge for electronic device applications will almost certainly be in the form of thin epitaxial films grown on cheaper and more abundant Si substrates. Integration on Si is doubly important due to the expectation that other Si components, such DRAM and non-volatile memory, will be included on the same chips [31]. As a consequence, perhaps the most significant hurdle to the widespread adoption of Ge in devices is the lattice mismatch with respect to Si. This problem is further aggravated by the thermal expansion coefficient mismatch in these two materials. Lattice mismatch is defined as:

$$\frac{\Delta a}{a}=\frac{a_{e}-a_{s}}{a_{s}}=\frac{a_{\mathrm{Ge}}-a_{\mathrm{Si}}}{a_{\mathrm{Si}}}=\frac{5.658-5.431}{5.431}=4.18\%$$ (1)

where *a*_e_ is the lattice parameter of the epitaxial layer and *a*_s_ is that of the substrate material. The lattice mismatch leads to a large buildup of strain energy in Ge layers epitaxially grown on Si. This strain energy is primarily relieved by two mechanisms: (i) generation of lattice dislocations at the interface (misfit dislocations) and (ii) elastic deformation of both the substrate and the Ge islands which form on the surface during early stages of growth (following the Stranski-Krastanow growth mode) [32]. In general, elastic deformation cannot accommodate all the strain and as a result, misfit dislocations nucleate at the island edges [33]. Many dislocations bend toward the growth direction and propagate, or thread, to the surface during growth. These threading dislocations severely degrade carrier transport properties by lowering carrier mobility and decreasing carrier lifetime. This dilemma necessitates the development of means to more gracefully bridge the lattice constants of the two materials. To accomplish this, several buffer architectures have been proposed and experimentally demonstrated. These approaches are summarized in Table 1. While not complete, Table 1 does highlight the wide variety of methods that have been explored for Ge on Si heteroepitaxy. Thinner buffers are generally preferred to reduce cost and facilitate heat dissipation. For FET applications, the buffer should be comprised of wide bandgap materials to (i) provide device isolation and (ii) suppress parallel conduction—two phenomena discussed in more detail later. A good buffer is further characterized by a low threading dislocation density (TDD) and low root-mean-square (RMS) roughness at the surface so that it may serve as a smooth template for active layer growth. It is believed that for device quality growth, a starting surface with a TDD of 10^6^ cm^−2^ or less, and a RMS roughness of 1 nm or less, is required. The approaches summarized in Table 1 generally fall into five categories: (i) direct epitaxy of Ge on Si, (ii) growing layers of Si_1–_*_x_*Ge*_x_* where *x* is graded from zero to one, (iii) surfactant mediated epitaxy (SME), (iv) aspect ratio trapping (ART), and (v) the oxide buffer. Each of these architectures is briefly reviewed in the following sections. It should be pointed out that buffer architectures demonstrating heterogeneous integration of GaAs on Si may also prove useful for Ge. This is due to the close lattice match between GaAs and Ge (0.07%), which significantly reduces the complexity of Ge on GaAs growth [34]. An excellent review of GaAs on Si epitaxy can be found in [35]. The higher bandgap of GaAs (1.42 eV) compared to Ge (0.67 eV) suggests that GaAs-based buffers should exhibit less parallel conduction than Ge-based ones. One exciting GaAs/Si work been added to Table 1 [25].

#### 2.1.1. Direct Epitaxy

The direct epitaxy approach is attractive due to its relative simplicity, and with the aid of high temperature hydrogen annealing, this method has demonstrated films with low RMS surface roughness (less than 1 nm) [38]. In regards to defect density however, direct epitaxy methods have had mixed results [47]. This method often involves a two-step low temperature then high temperature (LT/HT) Ge growth process. First a low temperature, low growth rate “seed” layer is grown which is intended to confine most of the defects. Together with Ge’s low surface diffusivity, lowering the growth temperature tends to prevent the impinging Ge from forming islands on the surface, resulting in flatter and more homogenous coverage of the Si substrate. After approximately 30 nm of growth the strain in the Ge surface will be fully relaxed due to the generation of misfit dislocations [48]. After the low temperature layer, a high temperature and high growth rate layer is deposited and is expected to be of higher crystalline quality since (i) the higher growth temperature annihilates defects and dislocations associated with strain relaxation, and (ii) the Ge is growing on a fully relaxed Ge “seed” layer instead of Si.

**Figure 2.**
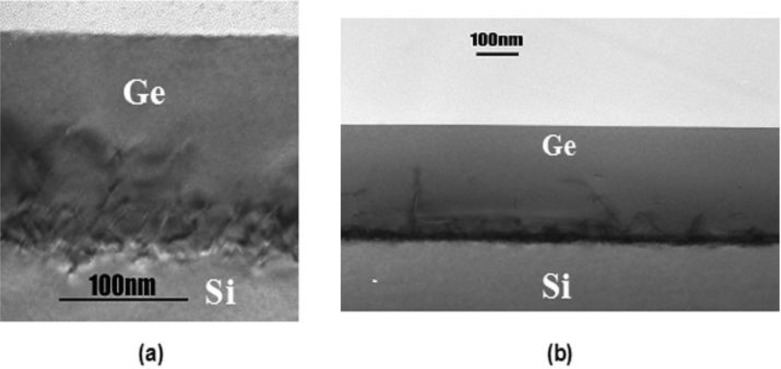
Cross-sectional TEM images of heteroepitaxial-Ge layers on Si; (**a**) 155 nm of Ge grown in single growth cycle with one H anneal step and (**b**) 400 nm of Ge layer grown in two growth cycles with two H annealing cycles (MHAH method), reprinted with permission from [36]. Copyright 2005, IEEE.

Separately from the two-step LT/HT approach, Nayfeh *et al*. [37] clearly demonstrated the benefit of hydrogen annealing of Ge layers on Si. The group reported an 88% reduction in RMS roughness for a 200 nm thick Ge layer grown on Si (at 400 °C) that was annealed for 1 hour at 825 °C in H_2_, compared to no reduction for a sample annealed in N_2_. They attributed the improved flatness to the increased surface mobility of the Ge-H cluster compared to Ge alone. Interestingly, the group found the ratio of optimal annealing temperatures for Si and Ge (1423 K/1073 K = 1.326) to be nearly equal to the ratio of their melting points (1687 K/1211 K = 1.393). In a separate paper [36] the same group used this approach to fabricate a Ge pMOSFET. They have grown a second Ge layer (400 °C, 250 nm thick) on top of a Ge layer produced by the method described in their previous work [37]. The resulting structure is shown in Figure 2. They observed a 2× increase in effective low-field mobility and a 3× increase in effective high-field mobility in a fabricated Ge pMOSFET compared to Si. Recently, this Ge on Si growth method was used in combination with a patterned SiO_2_ mask to achieve selective-area growth of Ge on Si and obtained a TDD of 1 × 10^7^ cm^−2^ and RMS roughness of 0.61 nm [49]. Hartmann *et al.* [50] reported a Ge surface with a TDD of 6 × 10^6^ cm^−2^ and RMS roughness of ~1 nm. The group used a similar two-step LT/HT growth process (400 °C/750 °C) and the total buffer thickness was 2.5 μm. Following growth, the structure was cyclically annealed (eight times 750 °C−10 min/900 °C−10 min) in H_2_. The hydrogen annealing was found to be critical for minimizing TDD, lowering it by nearly one order of magnitude (from ~4 × 10^7^ to 6 × 10^6^ cm^−2^), but at the cost of a small increase in RMS roughness (increasing it from ~0.7 to ~1.1 nm). It should be noted that the group has assumed TDD to be equal to pit density measured by atomic force microscopy (AFM). Kobayashi *et al.* [51] observed more than one order of magnitude difference between the TDD measured by plan-view transmission electron microscopy (TEM) and the pit density measured by AFM. Choi *et al.* [38] have grown multiple two-step LT/HT dual layer stacks and have used 30 min hydrogen annealing at 800 °C between each set. They found the AFM pit density reduced by 80% between the first and second sets of LT/HT layers. After three sets of growth and H_2_ annealing (and a total Ge thickness of ~1.81 μm) they achieved a TDD measured by plan-view TEM of 0.8–1 × 10^7^ cm^−2^ and RMS roughness of 0.4–0.6 nm. This is encouraging since additional sets of LT/HT growth and annealing could result in even lower TDD and RMS roughness, albeit with decreasing gains and additional buffer thickness.

Having a buffer composed of high bandgap material helps ensure that performance characteristics of a given device in an array are as much as possible independent of the on/off states of adjacent devices, or that is it electrically isolated from them. A pure Ge based buffer directly on Si would be expected to have poor device isolation characteristics due to the low bandgap of Ge. This alone is not necessarily mark against the direct epitaxy approach since it could be combined with commercially available silicon-on-insulator (SOI) substrates to improve device isolation, although this would also require that the buffer be quite thin to facilitate etching down to the buried oxide. A more serious concern for this approach is the prospect of parallel conduction of current through the buffer between the source and drain. An excellent review on the nature of parallel conduction can be found in [52]. The existence of additional higher conductivity paths between the source and drain, such as that provided by low bandgap buffer layers, has two primary consequences. First, it provides the means for a higher off-state current in FET devices, which in turn reduces the *I*_on_/*I*_off_ ratio and contributes power losses. This is exacerbated by the fact that the conductivity of these secondary paths is not effectively modulated by the gate voltage. Second, it undermines the device’s ability to confine carriers to the layer or path of highest mobility, which is particularly important in QWFET applications (discussed in section three). Failure to confine carriers to the highest mobility layer decreases on-state current, which also further reduces the device *I*_on_/*I*_off_ ratio. More investigation is needed to determine if parallel conduction is serious issue for the direct epitaxy approach.

#### 2.1.2. The Graded Si_1–_*_x_*Ge*_x_* Buffer

The graded Si*_x_*Ge_1–_*_x_* buffer approach aims to introduce the 4.2% lattice mismatch gradually rather than abruptly as in the direct epitaxy approach. This is possible since the lattice mismatch of Si_1–_*_x_*Ge*_x_* on Si is roughly proportional to the Ge composition (*x*). Furthermore, the threading dislocations created during growth of the initial low-Ge composition layers can be preserved and reused to relieve additional strain generated in subsequent higher-Ge composition layers by carefully controlling the grading rate and the growth temperature. The gliding of existing threading dislocations suppresses nucleation of additional dislocations. A notable implementation of this approach was carried out by Currie *et al.* [40]. The group achieved a remarkable TDD of 2.1 × 10^6^ cm^−2^. However, the total buffer thickness was 12 μm and the RMS roughness was 24.2 nm. Additionally, the group introduced a chemical-mechanical-polish (CMP) step near the growth midpoint since it was observed that the dislocations had a tendency to pile-up and become trapped, and therefore unable to continue to facilitate strain relief in subsequent layers. Samples with this added CMP step showed lower TDD of ~2.1 × 10^6^ cm^−2^ and lower RMS roughness of 24.2 nm (compared to 1–5 × 10^7^ cm^−2^ and 47 nm without the CMP). Loh *et al.* [53] later combined the graded SiGe buffer approach and the LT/HT approach to obtain an estimated TDD of 6 × 10^6^ cm^−2^ (estimated by etch pit density) and RMS roughness of 1.4 nm using a buffer of only about 160 nm.

To help address the concern of device isolation, the graded SiGe buffer approach could be carried out on silicon on insulator (SOI) substrates, as shown by the Loh group [53]. As with the direct epitaxy approach, more experiments are need to investigate to nuisance of parallel conduction.

#### 2.1.3. Surfactant Mediated Epitaxy (SME)

The amount of strain in epitaxial layers strongly influences the growth mode of the film. In the case of Ge on Si, the growth is flat (2D) for the first few monolayers. As the built-in strain energy increases with increasing film thickness, there is a sudden transition from 2D to 3D growth modes, also known as island growth [33]. It has been shown that this 2D-to-3D growth mode transition can be suppressed by deliberate introduction of a surfactant to alter the surface free energy, resulting in smoother layer-by-layer growth [54].

The general procedure for SME is as follows. The Si substrate is first capped with ~1 ML of the surfactant. The first surfactant investigated for Ge/Si growth was As. The extra valence electron from the As in the capping layer fills the dangling bonds at the Si surface, resulting in a stable, or energetically favorable termination. Following capping layer deposition, Ge growth begins. Surface termination with As remains energetically favorable since the Ge surface also contains dangling bonds. As a result, Ge atoms on the surface efficiently exchange sites with As in the capping layer. By this process the As capping layer “floats” on the surface even as Ge atoms continue to impinge on the surface, and the surface mobility of the growing species is strongly diminished, effectively suppressing the ability of the Ge to form islands. A drawback of the SME approach is that some of the surfactant inevitably incorporates into the film bulk, leading to a moderate (10^16^–10^19^ cm^−3^) n-type background doping.

In thicker films where islanding occurs, many defects thread towards the surface where the islands eventually coalesce. Using a surfactant to preserve the 2D growth mode also alters the defect structure that forms to relieve the lattice strain. LeGoues *et al.* [33] found that when As is used as the surfactant during Ge on Si(100) growth it results in V-shaped defects that can generate stacking faults and twins that extend throughout the entire film. Horn-von Hoegen *et al.* [55] showed that when Sb is used as the surfactant for Ge/Si(111) heteroepitaxy the threading defects generated to relieve stain during initial stages later self-annihilate as growth proceeds. This results in a network of dislocations confined at the Ge/Si(111) interface, and comparatively much fewer defects in the bulk of the film. A TDD of less than 10^8^ cm^−2^ and background doping of 3–4 × 10^19^ cm^−3^ were estimated. More recently, Wietler *et al.* [41] used Sb for Ge on Si(100) growth to achieve fully relaxed Ge layers with a TDD of 1.6 × 10^8^ cm^−2^ measured by plain-view TEM. The group used a relatively high substrate growth temperature of 670 °C and Sb flux of 2.4 ML/min. The high temperature helped facilitate Sb segregation to the surface, leading to a relatively low background doping of 3–4 × 10^16^ cm^−3^ in the Ge film. To further address the issue of surfactant incorporation in the Ge layer, it has recently been proposed to use carbon as the surfactant in place of Sb since carbon in Ge (i) does not act as a dopant and (ii) has very low solid solubility [56,57].

#### 2.1.4. Aspect Ratio Trapping (ART)

The ART approach, also called “epitaxial necking” [58], for Ge-on-Si heteroepitaxy is built on the premise that most threading dislocations do not run normal to the Ge/Si interface. Therefore, by growing Ge on Si within narrow trenches formed by patterned oxide, dislocations angled away from the normal are likely to eventually collide with and become “trapped” by the trench sidewall.

**Figure 3.**
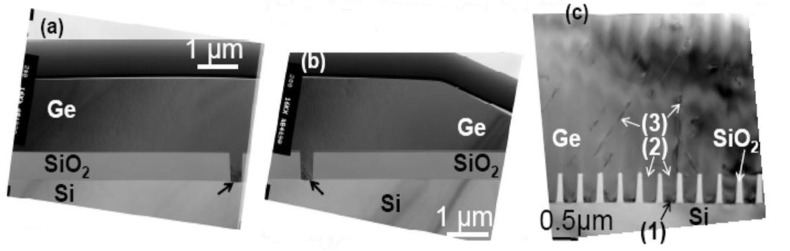
XTEM micrographs of Ge/Si heteroepitaxy using the ART method. (**a**,**b**) Uncoalesced Ge grown by epitaxial lateral overgrowth (ELO) from a single trench (indicated by a black arrow). The surface has been flattened using CMP; (**c**) ART Ge/Si in which growth from adjacent overgrown trenches has coalesced, reprinted with permission from [42]. Copyright 2009, The Electrochemical Society.

ART begins by thermally growing 0.5–1 μm of oxide on the substrate. Trenches are formed in the oxide using standard lithography and reactive ion etching (RIE). Bai *et al.* [59] developed a model to determine the optimal dimensions, or aspect ratio (*AR*=height/width), of the trenches. Park *et al.* [60] showed that trenches with *AR* > 1 were needed to effectively trap defects within the sidewalls. A drawback of ART growth is that, while the length of trenches is arbitrary, the width of the area available for Ge devices is restricted to less than 1 μm by the *AR* > 1 requirement and the need for relatively thin buffers. To obtain a larger area of high-quality Ge for device growth, the Ge can be overgrown beyond the trench height, resulting in epitaxial lateral overgrowth (ELO). However, when ELO emerging from adjacent trenches coalesces, it forms new defects, as shown in Figure 3c(3). Defects originating at the Ge/Si interface in the trench (1) do not appear to propagate beyond the trench height (2). To avoid the defects formed by ELO coalescence, Park *et al.* [42] later used a growth process optimized for lateral growth, and CMP, to develop long 20 μm wide regions of Ge, grown from a single trench, with a TDD of 1.6 × 10^6^ cm^−3^, shown in Figure 3a,b. For some device applications long 20 μm wide patches of high quality Ge may be sufficient [61]. If device area continues to improve, ART may become a viable method for creating Ge/Si virtual substrates for future high-performance Ge CMOS devices.

#### 2.1.5. The Oxide Buffer

Two distinctly different oxide-based approaches have emerged for heterogeneous integration of Ge on Si: (i) the epitaxial oxide buffer [46,62–64], and (ii) heteroepitaxial growth of Ge on high Ge content SiGe-on-insulator (SGOI) substrates [43,65–67].

**Figure 4.**
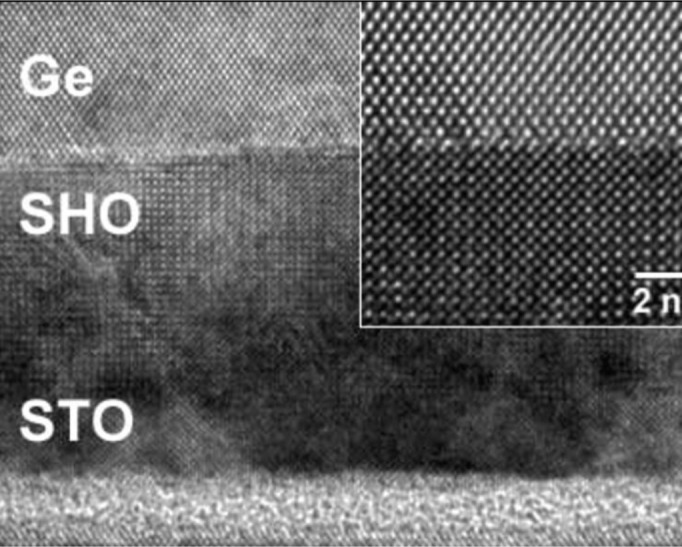
HRTEM image of Ge grown on SHO. The interface between Ge and SHO is atomically sharp and contains no interfacial layer (inset), reprinted with permission from [46]. Copyright 2007, Elsevier.

The epitaxial oxide based buffers are especially appealing due to (i) device isolation and (ii) the natural suppression of parallel conduction being inherently built-in to the structure. However, there is limited literature available on this method and more experiments are needed to clearly demonstrate its merit. Bojarczuk *et al.* [64] obtained a single-crystalline fully-relaxed layer (4 nm) of Ge on a (La*_x_*Y_1–_*_x_*)_2_O_3_/Si(111) template. Amorphous Ge was deposited on the crystalline (La*_x_*Y_1–_*_x_*)_2_O_3_ and subsequently heated to 450 °C under an Sb flux to induce an amorphous to crystalline transformation of the Ge. The Sb flux was found to reduce surface roughening during recrystallization. The quality of the Ge surface, in terms of RMS roughness and TDD, was not reported. Seo *et al.* [46] used a two stage SrHf*_x_*Ti_1–_*_x_*O_3_ buffer on Si(100) to obtain a continuous and flat epitaxial Ge film, shown in Figure 4. The group conveyed that the method needed improvement due to low Hall-mobility (100–300 cm^2^/Vs) and a high density of stacking faults and twin structures (10^9^ cm^−2^). Giussani *et al.* [63] used PrO_2_/Si(111) as a template for Ge(111) epitaxy and obtained a smooth single-crystalline Ge layer. However, an extended Ge deposition time was needed. Quantitative measurements of the surface quality were not provided.

**Figure 5.**
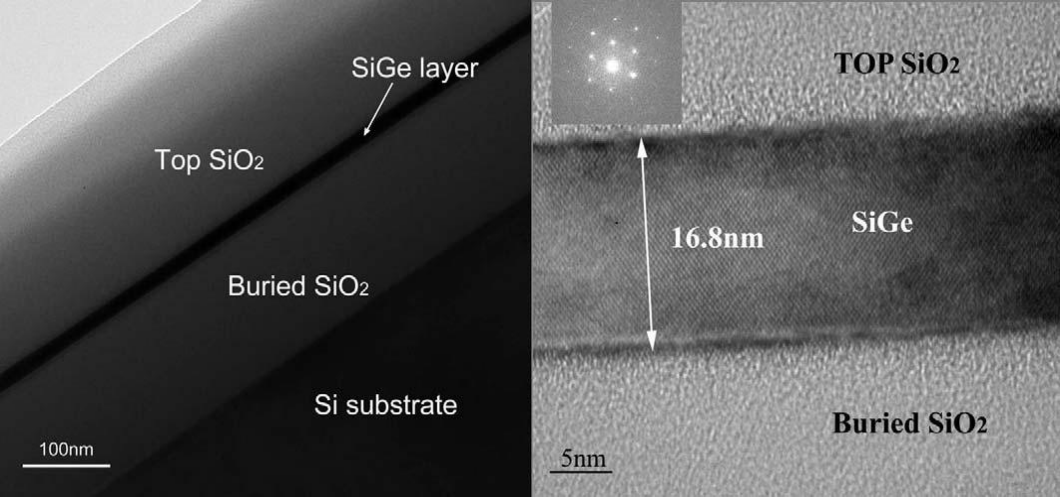
HRTEM images of Ge-rich SiGe layer produced by the Ge condensation technique. The Ge composition is 89%, reprinted with permission from [65]. Copyright 2009, Elsevier.

The second oxide based approach, Ge on high Ge content SGOI substrates, is relatively new but has produced encouraging results. The method usually involves using the Ge condensation technique, proposed by Tezuka *et al.* [67], to convert a standard SOI substrate into a SGOI substrate. In this technique, a layer of low Ge content Si_1–_*_x_*Ge*_x_* (*x* ≤ 0.15) is epitaxially grown on a SOI substrate. A thinner layer of Si is grown on this SiGe layer, and then the structure is cycled through oxidizing and annealing stages. Due to the preferential oxidation of Si over Ge [68], the original Si_1–_*_x_*Ge*_x_* layer recedes at the upper and lower bounds to leave behind SiO_2_. After oxidation and additional annealing in N_2_ to homogenize the Ge distribution, a thinner layer of Ge*-enriched* Si_1–_*_x_*Ge*_x_* is left sandwiched between thicker layers of SiO_2_ as seen in Figure 5. In the last step the top layer of SiO_2_ is etched with HF to reveal the crystalline Si_1–_*_x_*Ge*_x_* surface. Ma *et al.* [65] used an optimized oxidation and annealing sequence to achieve a Si_0.19_Ge_0.89_ (16.8 nm thick) surface with a RMS roughness of 0.8 nm and a TDD of 1.7 × 10^4^ cm^−2^. While the surface is not pure Ge, with the high Ge fractional content and exceptional surface quality it may prove to be a suitable template for Ge growth, or strained Ge in particular. Further experiments are needed to determine if this is the case. Due to the thick layer of SiO_2_ under the Ge rich Si_1–_*_x_*Ge*_x_*, devices grown on this platform should be well isolated. However, Ge rich Si_1–_*_x_*Ge*_x_* would be expected to have a bandgap near 0.8 eV, low enough to be a potential pathway for parallel conduction. Hutin *et al.* [8] showed this parallel conduction could be reduced by ensuring the Ge rich Si_1–_*_x_*Ge*_x_* layer is very thin (8 nm). Encouragingly, they reported an *I*_on_/*I*_off_ ratio of approximately 10^5^, the one of the best reported for a pMOSFET fabricated using the Ge condensation technique. Recently, even more encouraging results have been obtained [69].

On the topic of heterogeneous integration of Ge on Si substrates, there does not yet appear to be a clear winner for the best approach. However, many promising results have been obtained so far. The challenge of Ge on Si growth remains an important and ongoing area of research to unlock the potential of Ge based devices.

### 2.2. Developing a Suitable Gate Stack

The era of high-κ oxides has brought with it many exciting opportunities and challenges of its own. High-k materials deposited on Ge, in particular, have poised a special challenge due the higher reactivity of Ge (compared to Si) and the instability of the semiconductor’s native oxide, a subject discussed in more detail below. A number of excellent reviews which include a discussion of high-k oxides on Ge exist currently in the literature [2,11,27–29,70–73]. However, with the rapid pace of development in this area, recent advancements merit further review.

#### 2.2.1. Overview

The critical function of high-k oxides is to permit a greater *physical* thickness of dielectric material between the gate and channel (compared to SiO_2_) without sacrificing gate capacitance. The charge component, *Q*, of *I*_on_ is summoned by the combination of the gate voltage and gate capacitance, therefore a high gate capacitance is desired to increase *I*_on_. For decades this capacitance could be scaled higher by making the oxide thinner. However, there is a lower limit beyond which the oxide thickness cannot be reduced since tunneling current (leakage) increases exponentially with decreasing thickness. Oxides with a physical thickness less than ~2 nm exhibit a unacceptably large gate leakage current leading to excessive power dissipation [74]. The gate capacitance is:

$$C=\frac{\varepsilon_{0}k\;A}{t_{\text{ox}}}$$ (2)

where *A* is the area, *k* is the dielectric constant, is the permittivity of free space, and *t*_ox_ is the oxide thickness. The relation above motivates the implementation of high-k materials into gate stacks. The only way to increase *C*, when *t*_ox_ cannot be reduced further, is to increase *k* (assuming *A* is fixed).

An important fundamental concept in discussing gate stacks is equivalent oxide thickness (*EOT*). Since industry used SiO_2_ as the gate oxide for decades, researchers and device engineers have grown accustomed to using the thickness of the SiO_2_ as the primary metric to gauge the strength of the gate capacitance. Today, this remains the preferred means to assess gate capacitance, even after SiO_2_ is no longer the primary oxide used in today’s microprocessors. *EOT* is computed as:

$$t_{\text{eq}}=EOT=\left(\frac{3.9}{k_{\text{hiK}}}\right)t_{\text{hiK}}$$ (3)

where *k*_hiK_ and *t*_hiK_ are the dielectric constant and physical thickness of the high-k material respectively. For Ge and III-V devices, the ITRS calls for a manufacturable *EOT* of 0.68 nm by 2018 [31]. This is a good target for today’s research efforts. For a material with a dielectric constant of 20, this corresponds to a physical oxide thickness of 4.36 nm.

In truth, the description of the gate capacitance provided by Equations (2) and (3) is oversimplified. Today’s deep-submicron MOSFETs have entered a regime in which quantum mechanical effects and the gate electrode (degenerately doped poly-Si) depletion depth can no longer be neglected. These effects are reviewed in number of excellent works [74–76] and are not discussed in detail here. The thin depletion region in the poly-Si and the finite displacement of the charge centroid (in the channel) from the oxide interface both contribute an additional capacitance term appearing in series with the oxide capacitance. Therefore, the gate capacitance is more accurately modeled as:

$$\frac{1}{C}=\frac{1}{C_{\text{ox}}}+\frac{1}{C_{\text{poly}}}+\frac{1}{C_{\text{channel}}}.$$ (4)

Then the effective capacitance thickness (*ECT*, also called the electrical *EOT* in some literature) follows as a sum of three effective thicknesses as shown below:

$$ECT=EOT+t_{\text{poly}}+t_{\text{channel}}$$ (5)

where *t*_poly_ and *t*_channel_ are the effective SiO_2_ equivalent thicknesses of *C*_poly_ and *C*_channel_ respectively. These thicknesses can each contribute few Å to the *ECT. t*_poly_ can be reduced to about 0.5 Å by replacing the poly-Si gate electrode with a metal (which is itself separate technical challenge). However, *t*_channel_ is an intrinsic phenomenon, and likely intractable. For Ge FET devices, the ITRS calls for an *ECT* of 1.18 nm by 2018.

High-k materials can be deposited on Ge using a variety of methods. The most common are chemical vapor deposition (CVD) [77,78], physical vapor deposition (PVD) [79], molecular beam deposition (MDB) [80,81], and atomic layer deposition (ALD) (two variations: thermal [82], and plasma [83]). Among these four, ALD is the most popular since it has many advantages. In addition to allowing precise thickness control on the sub-nm scale, ALD offers superior step-coverage and conformity, which is doubly important as we move into the era of multi-gate and 3D transistor configurations [84].

Experimentally, poor performance has been obtained when high-k oxides are deposited directly onto the Ge channel due to a high density of defects that create electronic states in the bandgap. High-k materials intrinsically have a higher concentration of defects compared to SiO_2_ due to their higher coordination number. Bonds in high-k materials cannot relax and rebond at defect sites as well as SiO_2_. The concentration of defects, particularly those near the interface, has serious consequences for channel mobility. Charge trapped in the defect centers scatter carriers in the channel leading to degradation of channel mobility [74]. To address this issue, an interfacial layer (*IL*) is usually inserted between the high-k material and the channel. The *IL* material generally has a much lower dielectric constant and tends to form a higher quality interface (fewer defects) with the channel material. The reduction of the defect concentration at the interface is often called surface passivation and so these layers are also called passivating layers. The metric most commonly used to judge the quality of an interface is the interface trap density *D*_it_, which can be measured using methods reviewed in refs. [85,86] (the terms interface traps, interface charge, and interface states are all used interchangeably in the literature). *D*_it_ represents the number of trapped charges per cm^2^ per eV. The term eV appears since the energy levels of the traps are distributed across the bandgap. Therefore, trap density is reported as a function of energy level. Usually, however, only the density near the midgap is reported (unless otherwise stated).

In addition to interface passivation, a critical role of the *IL* is to put some distance between carriers in the channel and the high-k material. This is highly desirable since high-k materials have been shown to strongly impact channel mobility *even when not in direct contact with it* [87]. The reasons for this are not entirely clear, however three mechanisms have been put forward (listed without details here since they are beyond the scope this this article): remote optical phonon scattering (RPS) [88,89], remote Coulomb scattering (RCS) [90,91], and remote surface roughness scattering [92,93]. Whatever the mechanism, one thing is clear, the presence of a high-quality *IL* is critically important to preserving the intrinsically high carrier mobility of Ge in the channel. The importance of the *IL* channel-shielding effect must be carefully balanced with the *EOT* cost of including the *IL*. Since *IL*s have lower dielectric constants, they generally increase the total *EOT* presented by the high-k material and the *IL* in series. This is seen from the equation for adding capacitors in series (same form as in Equation (5)). Neglecting poly-Si depletion and quantum mechanical effects we have:

$$\frac{1}{C_{\text{eq}}}=\frac{1}{C_{\text{hi-k}}}+\frac{1}{C_{IL}}.$$ (6)

Combining Equations (2), (3), and (6) we can derive:

$$EOT_{\text{total}}=\left(\frac{3.9}{k_{\text{hi-k}}}\right)t_{\text{hi-k}}+\left(\frac{3.9}{k_{IL}}\right)t_{IL}$$ (7)

where *k_IL_* and *t_IL_* are the dielectric constant and physical thickness of the *IL* material respectively. To further illustrate the notion of the *IL EOT* cost, consider the following example. Consider an *IL* which is 1 nm thick and is comprised of material with a dielectric constant of 4.5. Assume at least 1 nm *IL* thickness is needed to give low *D*_it_ and good channel mobility. Since a physical thickness of greater than 2 nm is strongly desired to suppress gate leakage, 2 nm of high-k material with a dielectric constant of 20 is deposited on top of the *IL* for a total physical oxide thickness of 3 nm. This configuration gives a total *EOT* of 1.26 nm, approximately double the ITRS target indicated earlier.

The *EOT* cost of including an *IL* has motivated many experiments to find alternate passivation methods, usually wet chemical approaches that do not involve a physical layer of lower *k* material in the gate stack. These methods have generally been unsuccessful due to high *D*_it_ [94,95]. One of the primary goals of Ge gate stack research today is to simultaneously achieve low *EOT*, high channel mobility, and low leakage current. Such a device, however, has thus far proven to be exceedingly elusive. In fact, a general trend has been observed: as *EOT* is scaled down, peak channel mobility is degraded [11,74].

**Figure 6.**
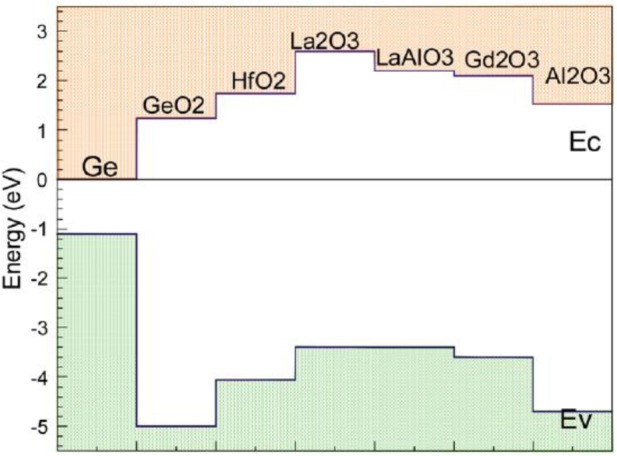
Calculated band offsets of oxides on Ge, reprinted with permission from [97]. Copyright 2013, AIP Publishing LLC.

Another important consideration in developing gate stacks is band offsets. Both the conduction and valence band offsets (CBO and VBO respectively) must be at least 1 eV in order to suppress Schottky emission of electrons or holes into the oxide bands (another source of leakage current) [96]. Figure 6 shows predicted Ge CBOs and VBOs with an array of common gate oxides. Almost always, the CBO is the smaller of the two offsets, so it is generally of greater interest.

The requirements from the gate oxide stack are summarized as follows:

1. Low *EOT* (should approach or exceed the targets set by the ITRS);
2. Low leakage current density (should not be higher than 1.5 × 10^−2^ A/cm^2^) [74];
3. Sufficient CBO and VBO (at least 1 eV for both);
4. Low *D*_it_ (less than 10^11^ cm^−2^·eV^−1^ is good, greater than 10^12^ cm^−2^·eV^−1^ is poor);
5. Minimally diminished channel mobility (although this is usually expected if *D*_it_ is low);
6. Sufficiently high dielectric breakdown electric field E_BD_ [73];
7. Thermodynamic and kinetic stability [74].

In regards to thermodynamic and kinetic stability, the parameters listed in requirements 1–6 should be relatively immune to high temperature device processing steps (for example if the gate oxide is amorphous it must not recrystallize during processing); 400 °C Ge MOSFET process technology has been demonstrated [98]. Following from this process, Ge gate stacks should be able to endure thermal budgets of at least 400 °C for 30 min. Although this is substantially less than typical thermal budgets for Si, it remains a serious challenge since (i) key high-k oxides, such as HfO_2_, are known to recrystallize at 400 °C [99], (ii) GeO_2_ (sometimes used as an *IL* for gate stacks on Ge) is highly unstable at this temperature (this is discussed in more detail in the next section), and (iii) Ge can interdiffuse with important high-k materials like HfO_2_ and ZrO_2_, even at temperatures as low as 300 °C, contributing to higher *D*_it_ values [71]. The most common materials used as high-k dielectrics on Ge are Al_2_O_3_, HfO_2_, and ZrO_2_. These materials have been selected since they have (i) relatively high *k* values as shown in Table 2, (ii) sufficient CBO and VBO, (iii) reasonable thermodynamic and kinetic stability, and (iv) adequately high breakdown electric field. Other materials are also being investigated such as Y_2_O_3_ [100], TiO_2_ [13], and La_2_O_3_ [101].

With the ground work covered, the following sections will review methods for implementing an *IL* on Ge surfaces. The role of the *IL* is twofold: (i) passivate the surface effectively (*i.e*., have low *D*_it_ and give undiminished channel mobility), and (ii) accomplish role (i) at the lowest *EOT* cost possible. The quality of the *IL* layer is determined by its ability to achieve the above stringent requirement. In the case of Ge, two *IL* approaches have emerged as the most successful over recent years: the GeO_2_ passivation layer [102–104], and the Si passivation layer [6,105–108]. Other approaches exist, such as surface nitridation [77], and sulfur passivation [109], however these approaches are not reviewed in this paper.

Before proceeding, a word of caution must be issued to the reader. One of the greatest issues plaguing gate stack research is the lack of consensus on the procedure for measuring and extracting *D*_it_ [85,86,110–113]. Without standardization of the *D*_it_ measurement, comparing outcomes for different gate stack approaches is difficult. This also increases the challenge in identifying a clear relationship between *D*_it_, channel mobility, and subthreshold slope.

#### 2.2.2. The GeO_2_ Passivation Layer

The success of Si native oxide SiO_2_ as an interface passivation scheme for Si devices helped sustain the growth of the microelectronics industry for decades. This has led many to investigate using Ge’s native oxide as a passivation layer in Ge devices. Ge’s native oxide is usually written as GeO*_x_*. The *x* is intended to emphasize that GeO_2_ is not always dominant (this is in contrast to Si where SiO_2_ is dominant). It is primarily composed of a mixture of GeO and GeO_2_ [114]. Prabhakaran *et al.* [115] showed that as annealing temperature is increased from 250 °C to 420 °C the GeO_2_ on the surface undergoes the following transformation:

$${\mathrm{GeO}}_{2}+\text{Ge}\to 2\mathrm{GeO}$$ (8)

Furthermore, at approximately 425 °C the GeO desorbs from the surface. This marks a stark contrast to the SiO_2_/Si interface in which the monoxide species is transformed to the dioxide species as annealing temperature approaches the oxide desorption temperature. The transformation is as follows:

$$2\mathrm{SiO}\to {\mathrm{SiO}}_{2}+\mathrm{Si}$$ (9)

Relations (8) and (9) illustrate the distinctly thermal decomposition pathways of native oxide layers on Si and Ge. Additionally, SiO_2_ does not desorb until ~760 °C. By comparison to the SiO_2_/Si interface, the GeO*_x_*/Ge interface is far less thermodynamically stable. Ge’s poor stability is exacerbated by the fact that GeO_2_ is soluble in water. This stark contrast in behavior helps explain why there is a substantial difference in quality (in terms of *D*_it_) between the SiO_2_/Si and the GeO*_x_*/Ge interface. For the SiO_2_/Si interface a *D*_it_ of ~10^10^ cm^−2^·eV^−1^ is routinely obtained [116]. However, for the GeO*_x_*/Ge interface, even 10^11^ cm^−2^·eV^−1^ is very difficult to achieve [117,118]. More recently, it has been found that GeO_2_ can serve as an effective *IL*, and the quality of the interface is strongly dependent on how the interfacial oxide is formed [102–104]. A wide variety of oxidation methods are being explored, including thermal oxidation [12,71,102,119–130], ozone or atomic oxygen exposure [131–136], high-pressure oxidation [103,104,137], radical oxidation [138], and plasma techniques [9,14,139].

Perhaps the greatest challenge of the GeO_2_ *IL* approach is controlling (i) the thickness of the GeO_2_, and (ii) the diffusion of Ge into high-k material. It is desirable to keep the GeO_2_ no thicker than is needed to passivate the interface (to minimize the *IL EOT* cost). This is made more difficult by the fact that Ge has a tendency to diffuse into the high-k material [103,140]; although some materials have shown to be more resistant to Ge up-diffusion and can even act as a barrier to it (Al_2_O_3_ is an example) [14]. Some works have observed that suppressing Ge interdiffusion with the high-k material results in improved performance [14,140]; suggesting defects are created during the interdiffusion process. Recently, researchers have shown some success in improving interface quality by *capping* the GeO_2_/Ge interface with diffusion resistant high-k material [13,14,138].

Recently, Zhang *et al*. [13] demonstrated an *EOT* of 0.65 nm using a gate stack of TiO_2_/Al_2_O_3_/Ge. The group reported their expectation that there were a few monolayers of GeO_2_ at the Al_2_O_3_/Ge interface. Previous analysis [141] of a device made using the same process found a *D*_it_ of about 3 × 10^11^ cm^−2^·eV^−1^ near the midgap. TiO_2_ is an attractive dielectric material since it has a very high dielectric constant of 80. However, it is rarely used since its CBO on Ge is almost zero. Al_2_O_3_ on the other hand has a relatively low dielectric constant of 9 and a CBO of 1.58 eV on Ge, which meets the 1 eV minimum to suppress gate leakage. For this work, a fair leakage current of about 1 × 10^−2^ *A*/cm^2^ at *V*_g_ = −1 V is reported for the combined TiO_2_/Al_2_O_3_/Ge stack. This is notable since a very poor leakage current might be expected due to the near zero CBO of TiO_2_ on Ge and the Al_2_O_3_ layer is fairly thin (~1 nm). Since it seems no single dielectric material can achieve everything required for an excellent gate stack, composite gate stacks provide some much needed flexibility, as well as many exciting and unexplored possibilities for Ge gate stack design.

**Figure 7.**
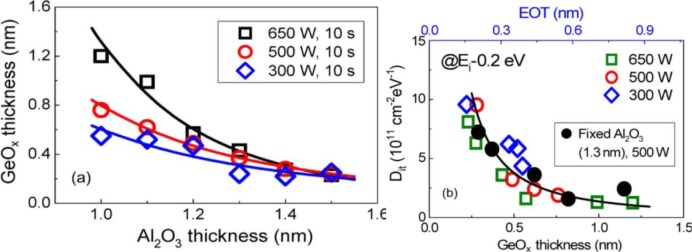
(**a**) Thicknesses of GeO*_x_* *IL*s with different Al_2_O_3_ thicknesses and plasma powers; (**b**) *D*_it_ of Au/Al_2_O_3_/GeO*_x_*/Ge MOS capacitors as a function of the GeO*_x_* *IL* thickness. The *D*_it_ reported is for 0.2 eV above the valence band, reprinted with permission from [14]. Copyright 2012, IEEE.

Another recent and promising development is post-plasma oxidation. Zhang *et al*. [14] demonstrated an *EOT* of about 1 nm and midgap *D*_it_ on the order of 1 × 10^11^ cm^−2^·eV^−1^ using a Al_2_O_3_/GeO*_x_*/Ge gate stack. What is unique about their approach is that the Al_2_O_3_ is deposited before the plasma oxidation. The plasma-oxidation process then grows the GeO*_x_* at the Al_2_O_3_/Ge interface. Therefore, the GeO*_x_* is confined from the very start. Since Al_2_O_3_ also acts as a diffusion barrier for oxygen, the group found that by controlling the thickness of the Al_2_O_3_ they could control the thickness of the GeO*_x_* interfacial layer. Additionally, the group reported a clear relationship between *D*_it_ and the thickness of the GeO*_x_*, shown in Figure 7. The group noted *D*_it_ increased sharply if the GeO*_x_* *IL* was reduced below 0.5 nm and also reported a high leakage current density of ~1 A/cm^2^, which is likely due to the relatively thin combined oxide thickness of ~2.5 nm.

#### 2.2.3. The Si Passivation Layer

The challenge of achieving high quality GeO*_x_*/Ge interfaces that are comparable to that of state-of-the-art SiO_2_/Si has inspired interest in the oxide/Si/Ge passivation scheme. In this approach, the Si is not always deliberately oxidized to form SiO_2_/Si/Ge. However, typically 2–3 ML of Si is consumed during processing to form a thin (~0.5 nm) layer of SiO_2_ at the Si surface [10,15]. The difficulty of overcoming the 4% lattice mismatch between Ge and Si has already been discussed at length. However, even extremely thin layers (less than 1 nm) of Si have been shown to passivate effectively and suppress the growth of Ge’s unstable native oxide [107]. This is below the critical layer thickness of Si on Ge, which has been reported to be between 1 and 2 nm [142]. The effectiveness of Si passivation has been shown to be sensitive to the Si growth parameters. It was found that diffusion of Ge into the Si layer was resulting in increased *D*_it_ and degraded device performance. Caymax *et al*. [6] showed this Ge diffusion could be suppressed with careful selection of Si precursors and the growth temperature, with lower temperatures helping to restrict diffusion.

**Figure 8.**
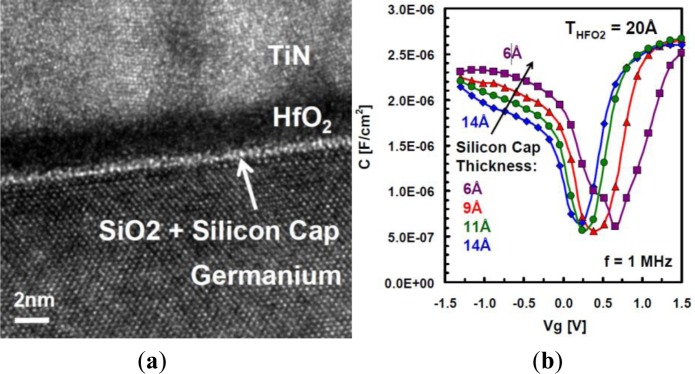
(**a**) Cross-sectional TEM image of a high-k metal gate stack with a thin Si cap on a Ge QWFET. Part of the Si cap is oxidized due to thermal cycles during the transistor fabrication process; (**b**) Capacitance-voltage characteristics of Ge pQWFETs with different Si cap thicknesses, reprinted with permission from [15]. Copyright 2010, IEEE.

Intel used Si passivation to demonstrate one of the best performing Ge PMOS planar devices to date [15]. A peak hole mobility of 770 cm^2^/V-s and equivalent oxide thickness of 1.45 nm was reported. Figure 8a shows a high resolution TEM image of the HfO_2_/SiO_2_/Si/Ge gate stack. A control sample in the Intel work without the Si *IL* showed more than a 10x reduction in mobility compared to the sample with a 0.6 nm Si cap. Figure 8b shows the capacitance voltage characteristics for Ge MOSFET samples with Si caps of different thicknesses. In addition to limiting carrier spill-out, thinning the Si cap from 1.4 nm to 0.6 nm also increases the gate capacitance in the inversion region. This is expected since thinning an *IL* comprised of a lower k material always reduces the *IL EOT* cost. Interestingly, the group found that increasing the Si cap thickness beyond 0.6 nm resulted in a reduction in peak hole mobility, even though it also resulted in a reduction of *D*_it_ from 9.0 × 10^11^ to 1.8 × 10^11^ cm^−2^·eV^−1^. This is due to insufficient VBO between the Si and Ge. The potential barrier to holes is not large enough to fully confine the carriers to the high-mobility Ge channel, resulting in carrier spill-out and a reduction in overall effective mobility. This presents a critical consideration in applying the Si passivation scheme for Ge. The Si thickness must be controlled on the angstrom scale to curb carrier spill-out. Considering one lattice constant of Si is 5.431 angstroms, controlling growth at this scale is quite challenging. This task is made more difficult since some portion of this Si layer is typically consumed in the formation of SiO_2_. This process must be carefully controlled as well to ensure a contiguous layer of crystalline Si remains on the Ge surface. The optimum thickness of this layer appears to be approximately 6–8 ML [10,15].

### 2.3. Achieving Low-Resistance Ohmic Contacts to n-type Ge

A key challenge in developing Ge-NMOS devices that perform comparably to the best Ge-PMOS devices today, is obtaining low-resistance ohmic contacts to n-type Ge. Low-resistance contacts are essential for a high drive current *I*_on_. Interface states can have either donor-like or acceptor-like behavior. Depending on this behavior, these interface states can pin the Fermi level at the metal/semiconductor interface. For a helpful and informative review of Fermi energy pinning see [23]. By inserting an ultrathin dielectric layer between the metal and the n-doped Ge, the Fermi level can be unpinned. Fermi-level pinning close to the Ge valence band edge at the metal/Ge junction [143,144] results in a large electron Schottky barrier, which translates to a high specific contact resistivity to n-type Ge. It has been demonstrated that thin potential barriers with low CBO on Ge could enable a high tunneling current. By deliberately facilitating a high tunneling current, the effective contact resistance at the junction is reduced. This is the primary mechanism behind the metal-insulator-semiconductor (MIS) contact to Ge [145]. The insertion of thin potential barriers, such as that provided by thin layers of Al_2_O_3_ [146], SiN_3_ [147], TiO_2_ [145,148], ZnO [24], Ge_3_N_4_ [149], GeO*_x_* [150,151], MgO [152,153], and Y_2_O_3_ [154] have been shown to reduce the Schottky barrier height as well as facilitate the unpinning of Fermi-level in n-type Ge.

It has been recently reported that the lower CBOs obtained for the crystallographic oriented TiO_2_/Ge system, irrespective of the Ge crystallographic orientation, presents a potential for employing a TiO_2_ insulating layer for MIS contact applications. Crystallographic-orientation agnostic TiO_2_-based MIS contacts may be particularly useful in the next generation of Ge FinFETs, where different Ge orientations can be exploited to facilitate mobility enhancement for *n*- and *p*-channel devices [148].

## 3. Opportunities

### 3.1. A Comparison: Ge versus III-V for PMOS

**Figure 9.**
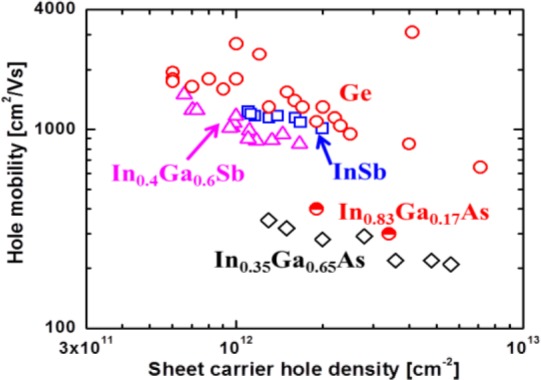
Room temperature hole mobility *vs.* sheet carrier density in biaxially compressed QWs: InSb [157,158], In_0.4_Ga_0.6_Sb, In_0.35_Ga_0.65_As [189,190], In_0.83_Ga_0.17_As [191], Ge [15,192–197].

High-performance n-channel InGaAs quantum well field effect transistor (QWFET) on Si has been demonstrated [25,26,155,156]. However, the demonstration of an equally high-performance *p*-channel QWFET within the same material system, which is needed for energy efficient complementary logic circuits, remains elusive till date due to low μ_h_ in InGaAs. Strained III-Sb materials (*i.e*., InSb [157,158], InGaSb [159–167], and GaSb [160,165,168–173]) are potential *p*-channel candidates due their high hole mobilities. These materials have low in-plane heavy-hole effective mass when applying a biaxial compressive strain of 1%–2%. By comparison Ge has much higher bulk hole mobility (μ_h_ =1900 cm^2^/Vs) even without strain. A μ_h_ higher than 2400 cm^2^/Vs was achieved for Ge using biaxial strain, as shown in Figure 9. Theoretical investigations of hole transport in 1.5%–2% biaxial strained III-V semiconductors show an increase in μ_h_ up to a factor of 2 over unstrained value may be possible [174–178]. However, μ_h_ in 2% biaxial-strained *Ge* can be increased up to 4000 cm^2^/Vs [179–181], which is significantly higher than any III-V materials. Considering several material choices and strain engineering in the channel, Ge is the best choice to obtain high μ_h_ *p*-channel devices that can be heterogeneously integrated on Si. In order to realize a Ge QWFET configuration, high bandgap barrier layers are essential in order to (i) eliminate parallel conduction to the channel [25,26,155,156]; (ii) provide a large VBO for hole confinement; (iii) achieve a high-quality high-k/barrier interface [182–185]; (iv) control the lattice mismatch [25,186]; (v) provide strain to the active channel; (vi) give superior interface properties; and (vii) improve ohmic contacts [187,188].

**Figure 10.**
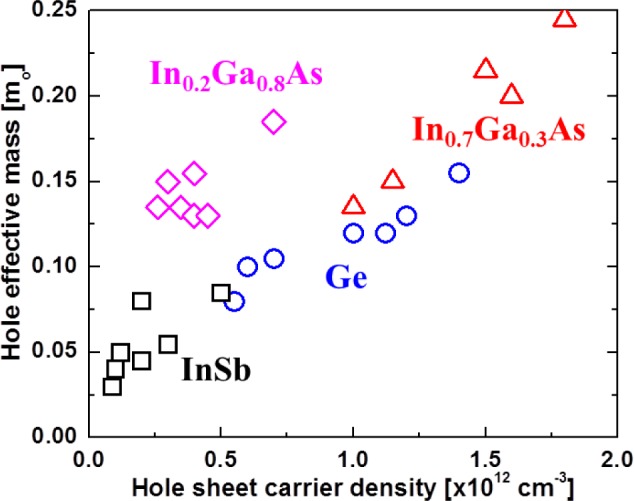
Effective mass *vs*. sheet carrier density in biaxially compressed QWs.

The biaxial compression in the channel causes splitting of the heavy hole (*hh*) and light hole (*lh*) valence bands, and causes the in-plane *hh* mass to behave as a light hole. This results in an increase in μ_h_ by reduction of *hh* mass. By utilizing biaxial compression during the growth of *p*-channel device structures, high μ_h_ (at 300K) in antimonide QWs has been achieved. μ_h_ is reported as a function of sheet carrier density (*N*_s_). In GaSb, a μ_h_ of 1350 cm^2^/Vs was reported (at *N*_s_ = 1.1 × 10^12^ cm^−2^ with 1% strain) [168]. In InGaSb, a μ_h_ of 1500 cm^2^/Vs was reported (at *N*_s_ = 7 × 10^11^ cm^−2^ with 2% strain) [198]. In InSb, a μ_h_ of 1230 cm^2^/Vs was reported (at *N*_s_ = 1.1 × 10^12^ cm^−2^ with 2% strain) [157]. These works all used GaAs substrates. The reported values are far below the theoretical predictions by Hinckley and Singh [175]. Moreover, these are lower than the μ_h_ of 2700–3100 cm^2^/Vs (at *N*_s_ = 0.5–3 × 10^12^ cm^−2^) that is reported for Ge QW devices, as shown in Figure 9 [192–197]. In addition, the μ_h_ for arsenide-based materials (InGaAs) is much lower than for Ge or Sb-based QWs [15,173,189–191]. Figure 10 shows the in-plane *hh* effective mass as a function of *N*_s_ in biaxially compressive strained QWs from Shubnikov-de Haas or cyclotron resonance measurements at low temperature for several materials. These results strongly suggest that the best *p*-channel material is either InSb or Ge. Utilizing Ge, with its lower lattice mismatch to Si (InSb is ~19% *versus* ~4% for Ge), as the channel material in a QWFET configuration appears to be the most attractive option for high-mobility low-power PMOS logic.

### 3.2. Ge Device Architectures

**Figure 11.**
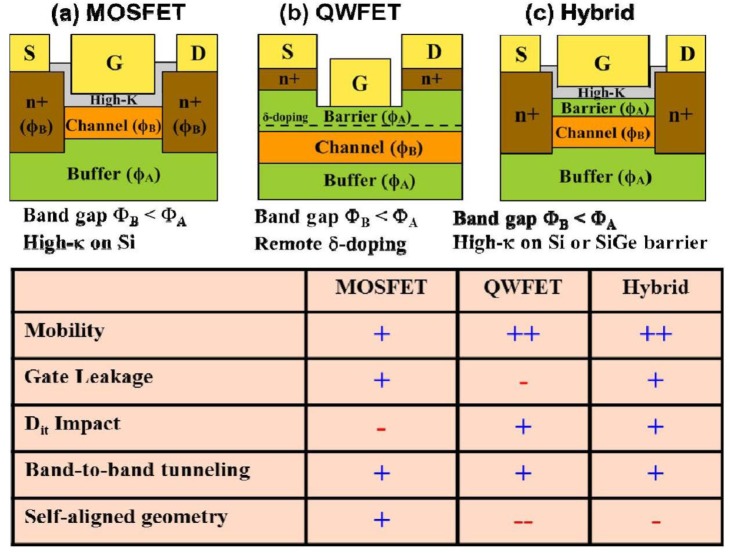
Device research vehicle for Ge on Si for low-power logic, and comparison of MOSFET, QWFET, and MOS-QWFET structures.

In the post-Si CMOS era, Ge is attractive due to its 2× higher μ_n_ and 4× higher μ_h_ (compared to Si), and its lower bandgap allows for better supply voltage scalability. However, a lower supply voltage results in (i) a lower operating electric field which then lowers carrier velocity (resulting in lower *I*_on_), and (ii) a lower gate voltage swing (effecting the transistor’s ability to switch off and resulting in increased leakage power). Therefore, new materials and innovative device architectures are required to continue transistor miniaturization. Figure 11 shows the Ge device research vehicle and compares three different device architectures: (i) conventional MOSFET, (ii) the QWFET, and (iii) metal-oxide quantum well FET (MOS-QWFET). MOS-QWFETs are a hybrid architecture in which an oxide layer replaces the upper barrier, or is deposited on top of the upper barrier, in the QWFET architecture. In the conventional MOSFET, the oxide is directly on the channel, and gate electrode is on top of the oxide. Mitard *et al*. [10] demonstrated a conventional Ge pMOSFET with a gate length of 65 nm, good *I*_on_/*I*_off_, and scalable *EOT*.

The QWFET architecture employs a modulation doped ultra-high mobility Ge channel which facilitates efficient carrier transport at high velocity (*υ*(*x*) = μ × *E*(*x*)) between the source and the drain, even at very low electric field. The channel is sandwiched by large bandgap barrier materials (SiGe or Si) to reduce the leakage. In this case, the Ge channel is biaxially compressive-strained by the barrier layers. The barrier layers must also provide a VBO sufficiently high to confine holes inside the Ge channel. A modulation doping layer, separated by spacer or barrier layer, eliminates the ionized impurity scattering with holes inside the Ge channel. Therefore, the Ge channel itself is undoped, and the amount of modulation doping is carefully selected such that no charge exits underneath the gate in the absence of voltage applied to gate electrode. The important parameters in designing Ge QWs to enhance hole mobility are (i) the percentage of strain induced in the Ge channel, (ii) the VBO between the narrow bandgap Ge channel and the wide bandgap SiGe buffer (which determines the maximum number of holes confined in the QW), (iii) the number and effective mass of the carriers in the *lh* and *hh* bands, and (iv) the energy split between the *hh* and the *lh* bands. Using this approach, high performance Ge pQWFETs have been demonstrated at low supply voltage of −0.5V [15].

There are two varieties of QWFET architecture commonly employed in the device research community: normal and inverted. In the normal QWFET the modulation doping layer is placed above the channel. In the inverted QWFET this doping layer is placed underneath the channel. The planar (not Fin-FET or multigate design) Ge pQWFET, though it has been demonstrated, still falls short of expectations due to large separation between the source and drain metal electrodes [15]. To fabricate ultra-scaled devices below sub-22nm gate length with improved short-channel performance several issues must be overcome, namely, (i) the 1:1 ratio between the lateral to vertical scaling, (ii) the reduction in upper barrier thickness (Φ_A_) (need to be less than 2 nm), and the (iii) reduction of source-to-drain spacing. The reduction in upper barrier thickness reduces the barrier resistance, which in turn improves (i) *I*_on_, (ii) switching speed, and (iii) the delay of the transistor. However, the reduction in upper barrier thickness results in increased gate leakage current for Schottky-gate (no oxide layer) QWFETs, shown in Figure 11b. The gate leakage current can be overcome with the integration of a high-k dielectric oxide, as discussed in earlier, resulting in the MOS-QWFET architecture, shown in Figure 11c, although the distinction between the QWFET and MOS-QWFET is not usually made. MOS-QWFETS exhibit significantly decreased *EOT* and reduced gate leakage (*J*_G_) [15]. Therefore, the combination of the QWFET structure and the high-k gate dielectric is currently the best option for low-power logic (supply voltage of −0.5 V). In order to implement Ge MOS-QWFETs in future high-performance logic applications, scalability (both in terms of physical gate length and the overall footprint) is mandatory. A thinner upper barrier is needed for ultra-scaled buried-channel devices. Due to this stringent requirement, the modulation doping layer sometimes placed underneath the channel, resulting in the inverted QWFET structure. This results in a more scalable device with faster switching, lower dynamic power consumption and minimized *I*_off_ [15].

### 3.3. State-of-the-Art Ge MOSFETs and MOS-QWFETs

**Figure 12.**
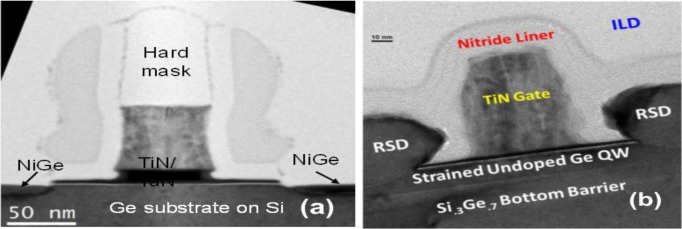
**(a)** Cross-sectional TEM micrograph of a 65 nm Ge- pMOSFET, reprinted with permission from [10]. Copyright 2008, IEEE; (**b**) Cross-sectional TEM micrograph of a Ge pQWFET, reprinted with permission from [15]. Copyright 2010, IEEE. Note the raised source/drain (RSD) in the QWFET which allows for reduction/removal of the S/D implantation.

**Figure 13.**
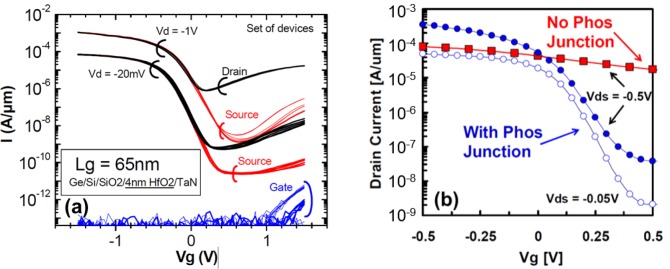
Transfer characteristics of measured (**a**) 65 nm Ge pMOSFET at low and high V_DS_ with superior reproducibility, reprinted with permission from [10]. Copyright 2008, IEEE; (**b**) 100 nm Ge pQWFET at *VDS* = −0.05 V (open circle) and −0.5 V (solid circle). The device exhibits a subthreshold slope (SS) of 97 mV/dec enabled by the phosphorus junction layer underneath the channel, which suppresses parallel conduction through the SiGe buffer, reprinted with permission from [15]. Copyright 2010, IEEE.

**Figure 14.**
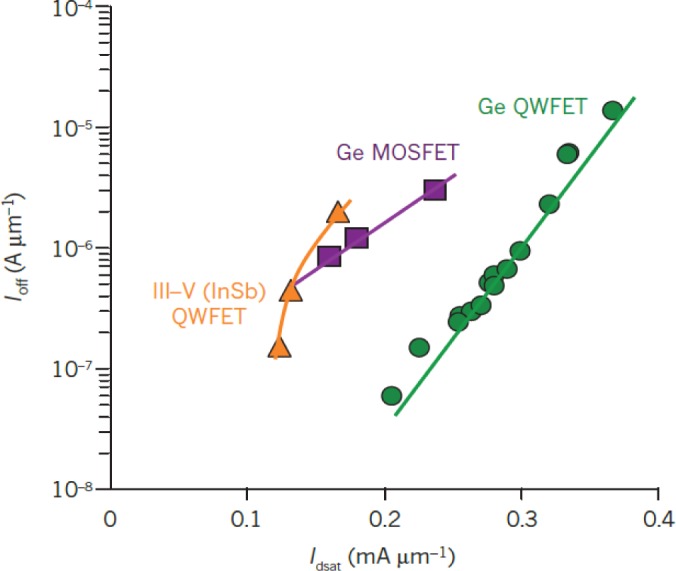
Benchmarking relation (*I*_off_ *vs. I*_dsat_) of 65 nm Ge pMOSFET, 40 nm InSb QWFET and 100 nm Ge QWFET at a supply voltage of 0.5 V. The Ge QWFET demonstrated the highest ON current at a given OFF-state leakage current, reprinted with permission from [2]. Copyright 2011, Nature Publishing Group.

Figure 12a,b show the cross-sectional TEM micrograph of a conventional surface channel 65 nm Ge pMOSFET [10] and the Ge pMOS-QWFET with in-situ boron-doped SiGe raised source/drain [15], respectively. Figure 13a shows the transfer characteristics of a 65 nm Ge pMOSFET at low and high *V*_DS_ with superior reproducibility [10]. Figure 13b shows the transfer characteristics for a 100 nm Ge QWFET at *V*_DS_ = −0.05V (open circle) and −0.5V (solid circle) [15]. The QWFET device exhibits a subthreshold slope (SS) of 97 mV/dec enabled by the phosphorus junction layer underneath the channel, which suppresses parallel conduction through the SiGe buffer as reported in [15]. Figure 14 compares saturation current (*I*_dsat_, also called *I*_on_) *vs*. off-state leakage current (*I*_off_) characteristics of 65 nm Ge pMOSFET [10], 40 nm InSb QWFET [157], and 100 nm Ge MOS-QWFET [15] at supply voltage of −0.5 V. The Ge MOS-QWFETs exhibited 2 × *I*_dsat_ for the same *I*_off_, demonstrating its potential for PMOS on a Si substrate. However, shorter gate length (<50 nm) Ge QWFETs in a planar configuration will be a significant challenge due to the self-aligned gate architecture needed for scalable enhancement mode devices for low power and high-speed digital logic applications.

**Figure 15.**
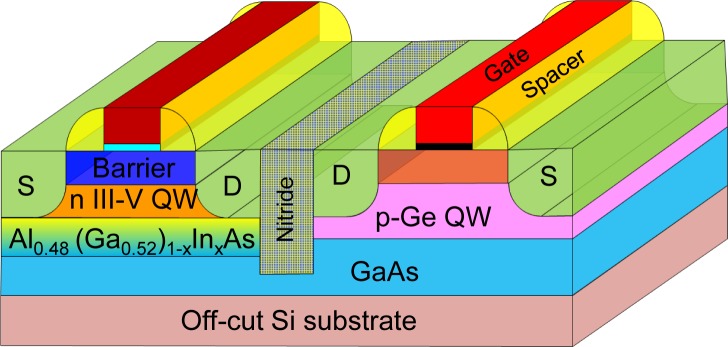
Schematic of extremely-high mobility NMOS and PMOS co-integrated for complete CMOS heterogeneously integrated on Si.

Furthermore, a list of well-established device metrics used for benchmarking logic transistors is essential [199]. These includes (a) CV/I delay as a function of transistor channel length for studying intrinsic speed of the proposed technology, (b) energy-delay product as a function of transistor channel length for optimizing switching energy and power consumption, (c) SS and drain-induced barrier lowering behavior as a function of transistor channel length which is critical for establishing the scalability, and (d) intrinsic delay CV/I *vs. I*_on_/*I*_off_ ratio to understand the performance and leakage tradeoff. At present, *p*-channel Ge MOS-QWFETs show great promise. Co-integration with *n*-channel InGaAs based MOS-QWFETs, as shown in Figure 15, offers an attractive pathway to extreme-high mobility CMOS. Ge based FinFET technology has recently been demonstrated [69,200]. Very recently, a Ge FinFET device obtained record *I*_on_/*I*_off_ characteristics with a highly scaled gate length and *EOT* of 35 nm and 0.8 nm respectively, representing a considerable breakthrough [200].

## 4. Conclusions and Outlook

In summary, germanium based *p*-channel devices are beginning to show signs of readiness for production, especially in light of recent breakthroughs [200]. Ge *n*-channel devices continue to lag behind, but are also making progress due to improved ohmic contacts [24,145]. The dual-Ge path for post-Si CMOS is becoming an increasingly viable option. However, to achieve the highest performance possible, the III-V/Ge path (NMOS/PMOS) is the most attractive (as shown in Figure 15), although it comes at the cost of increased processing complexity.

Five categories of buffer architectures for heterogeneous integration on Si have been discussed. Among them, the Ge condensation method has produced promising initial data, but now must demonstrate suppression of parallel conduction, and high *I*_on_/*I*_off_ ratios at scaled gate lengths. The low-temperature/high-temperature method combined with hydrogen annealing has also shown good results and now must also demonstrate that parallel conduction can be controlled to achieve low I_off_ currents and high *I*_on_/*I*_off_ ratios. The SME method also offers an interesting alternative approach for Ge/Si heteroepitaxy, especially if the matter of unintentional background doping can be resolved. The ART approach has been shown to produce narrow regions of excellent quality Ge, and has already been used in a commercial product [61].

Ge gate stacks have made tremendous strides thanks to many years of international collaborative effort. Two passivation schemes have emerged from over a decade of research. GeO*_x_* passivation has shown it can deliver interfacial layers with *D*_it_ comparable to today’s state-of-the-art Si devices. These methods must now (i) be demonstrated at scaled gate lengths, and (ii) focus on improving the thermodynamic stability and reliability characteristics of these gate stacks. The approach of capping the GeO*_x_* with a layer of diffusion-resistant high-k material is an excellent step in achieving this.

The metal-insulator-semiconductor (MIS) approach has shown it can effectively reduce the contact resistance to n-type Ge. These n-Ge low resistance ohmic contacts are needed to develop Ge NMOS devices which can perform on par with Ge PMOS devices already established. If comparably performing Ge NMOS and PMOS can be obtained at scaled gate lengths it unlocks the dual-Ge CMOS path, which will substantially simplify device processing procedures (resulting in reduced costs).

The advantages of Ge over III-V for *p*-channel devices have been reviewed. As a result of many years data-driven analysis, there appears to be strong consensus in the device research community that Ge is the best alternative for PMOS devices (if current strained-Si PMOS technology stalls). InSb appears to be the best III-V alternative, however its lattice mismatch with Si is 19%, requiring more complex and expensive buffer architectures compared to Ge (4% lattice mismatch).

Ge device architectures have also been reviewed, along with important design considerations for Ge MOSFETs and QWFETs. The hybrid architecture, the MOS-QWFET, shows clear advantages in terms of higher *I*_on_ and lower *I*_off_. However, these devices are also difficult to scale down to gate lengths of less than 50 nm due to the challenge of developing a self-aligned gate. New Ge FinFET devices have shown they can overcome this limitation.

## Figures and Tables

**Table 1.** Summary of buffer architectures for Ge epitaxy on Si-based substrates.

Category	Approach	Details	Substrate	Bandgap of buffer material (eV)	Total thickness (nm)	Ref.
Direct Epitaxy	Multiple hydrogen annealing for heteroepitaxy (MHAH)	direct epitaxy of Ge; multiple cycles of growth (400 °C) and high temperature hydrogen annealing (700–800 °C)	standard bulk Si	0.67	400	[36,37]

	Multiple cycles of low temp, high temp, hydrogen annealing	(i) deposit Ge directly on Si at low temp; (ii) deposit Ge at high temp onto low temp layer; (iii) high temperature hydrogen annealing; (iv) repeat (i) to (iii) 3 or 4 times	Si(100) 4° offcut towards <110>	0.67	2400 (4 cycles) 1400 (3 cycles)	[38]

Graded SiGe Buffer	Step graded Si_1–_*_x_*Ge*_x_* buffer	0.8 um Si_0.1_Ge_0.9_, followed by 0.8 um Si_0.05_Ge_0.95_, followed by 1 um Ge (all at 400 °C); 750 °C annealing between layers.	Si (100) 6° offcut towards <110>	0.67–1.12	3600	[39]

	Step graded Si_1–_*_x_*Ge*_x_* buffer	(i) 0.5–1.0 um Si_0.7_Ge_0.3_ layer grown on Si; (ii) 0.3 to 1.0 um Si_0.3_Ge_0.7_; (iii) 10–50 nm phos. doped Si_0.3_Ge_0.7_ to suppress parallel conduction; (iv) 0.5–3.0 um Si_0.3_Ge_0.7_.	Si	0.67–1.12	~1550 (est. from TEM image)	[15]

	Continuously Graded Si_1–_*_x_*Ge*_x_* and chemical-mechanical polishing (CMP)	(i) Graded from Si to Si_0.5_Ge_0.5_ at 10% Ge um^-1^; (ii) 1.5 μm *Si*_0.5_*Ge*_0.5_ cap; (iii) top 5 μm removed by CMP; (iv) Si_0.5_Ge_0.5_ graded to Si_0.08_Ge_0.92_ at 10% Ge um^−1^; (v) 1.5 um Ge cap.	Si(100) 6° offcut towards <110>	0.67–1.12	12000	[40]

Surfactant-Mediated Epitaxy	A surfactant (Sb) is used to alter the strain-relief mechanism in Ge on Si epitaxy	One monolayer (ML) of Sb is deposited on the Si substrate followed by 1 μm of Ge; a high Sb flux of ~2.4 ML/min is maintained during Ge growth.	Si(100)	0.67	~1000	[41]

Aspect Ratio Trapping	ART combined with epitaxial lateral overgrowth (ELO) and CMP	Ge is grown in patterned trenches in thermally grown SiO_2_; dislocations terminate on trench sidewalls; lateral Ge growth begins beyond trench height; CMP used to flatten the surface.	Si(100)	0.67	~1000	[42]

Oxide Buffer	Ge condensation technique	Epitaxial SiGe layers are grown on silicon-on-insulator (SOI) substrates followed by successive thermal oxidation.	strained and unstrained SOI	not reported	not reported	[43]

	Epitaxial SHTO buffer	(i) Epitaxial SrTiO_3_ is deposited using method in ref. [44]; (ii) epitaxial SrHf*_x_*Ti*_x_*_-1_O_3_ grown; (iii) Ge grown in two steps, 610 °C for nucleation, then 350 °C for homogenous coverage.	Si(100)	3.5 (STO) 6.5 (SHO) [45]	~ 20 (est. from TEM image)	[46]

GaAs Buffer	GaAs nucleation and buffer layer	An InGaAs QWFET heterogeneously integrated on Si was demonstrated using this buffer.	Si(100) 4° offcut towards <110>	1.42	500	[25]

**Table 2.** Static dielectric constant *k* and experimental bandgap for gate dielectrics common in the literature (reproduced from ref. [74]).

*k*		Gap (eV)
SiO_2_	3.9	9
Si_3_N_4_	7	5.3
Al_2_O_3_	9	8.8
Ta_2_O_5_	22	4.4
TiO_2_	80	3.5
SrTiO_3_	2000	3.2
ZrO_2_	25	5.8
HfO_2_	25	5.8
HfSiO_4_	11	6.5
La_2_O_3_	30	6
Y_2_O_3_	15	6
α-LaAlO_3_	30	5.6
